# Patients suffering from psychological impairments following critical illness are in need of information

**DOI:** 10.1186/s40560-019-0422-0

**Published:** 2020-01-09

**Authors:** Johan H. Vlake, Michel E. van Genderen, Anna Schut, Martijn Verkade, Evert-Jan Wils, Diederik Gommers, Jasper van Bommel

**Affiliations:** 1 Department of Intensive Care, Erasmus Medical Centre, Room Ne-403, Doctor Molewaterplein 40, 3015 GD Rotterdam, the Netherlands; 2 Department of Intensive Care, Franciscus Gasthuis & Vlietland Hospital, Rotterdam, the Netherlands; 3 Department of Intensive Care, Ikazia Hospital, Rotterdam, the Netherlands; 4 Department of Intensive Care, IJselland Hospital, Rotterdam, the Netherlands

**Keywords:** Intensive Care, Critical Illness, virtual reality, Posttraumatic stress disorder, Post-Intensive Care Syndrome (PICS)

## Abstract

**Background:**

Because critical illness survivors frequently experience several long-term psychological impairments altering quality of life after ICU, there is a trend towards increasing follow-up care, mainly via ICU follow-up clinics. Despite these and other initiatives, understanding of patient’s post-ICU needs to help them cope with their problems and subsequently improve quality of life is largely lacking. Our aim was therefore to assess the needs, expectations and wishes in ICU survivors to receive information with the purpose to help them better grasp ICU treatment. In addition, we assessed the perceived burden of psychological trauma after ICU treatment and the health-related quality of life (HRQoL) up to 2.5 years after ICU discharge.

**Methods:**

In a multicentre, retrospective cross-sectional cohort study, the needs and preferred intervention methods were assessed using a self-composed inventory in adult mechanically ventilated ICU survivors (*n* = 43). Additionally, the Impact of Event Scale Revised, the Beck Depression Inventory, the EuroQol-5D-5L, and the Short-Form 12 were used to assess psychological burden and HRQoL.

**Results:**

A substantial proportion of all ICU survivors (59%, 95% CI 44% to 74%) suffered from psychological impairments after ICU treatment. Seventy-five percent of these patients expressed a wish to receive information, but only 36% desired to receive this information using a commonly used information brochure. In contrast, 71% of these patients had a wish to receive information using a video film/VR. Furthermore, only 33% of these patients was satisfied with the information provided by their treating hospital. Patients with psychological PICS reported a worse HRQoL as compared to a normative Dutch sample (*P* < 0.001) and as compared to patients without psychological PICS (*P* < 0.01).

**Conclusions:**

In a Dutch cohort of critical illness survivors, a substantial part of ICU survivors suffer from psychological impairments, such as PTSD and depression, which was associated with a worse HRQoL. These patients are in need of information, have no desire using an information brochure, but are willing to receive information using a video film/virtual reality module. These results support the exploration of such an intervention.

## Background

Health-related quality of life (HRQoL) after ICU discharge is increasingly becoming the focus of intensive care medicine rather than intensive care unit (ICU) survival alone. Due to advances in critical care medicine, we are confronted with a growing population of ICU survivors suffering from long-term impairments which are challenging to cope with [1–5]. These post-ICU impairments, collectively referred to as the Post-Intensive Care Syndrome (PICS), consist of psychological, physical, and cognitive impairments [3, 6]. Depression and posttraumatic stress disorder (PTSD) related symptoms are the main components of psychological PICS. Psychological PICS is considered the most important component associated with patient-reported unacceptable outcome and decreased HRQoL [3, 7]. Because these psychological sequelae can persist up to 5 years after ICU discharge, treatment of psychological PICS sequelae is becoming an important target to improve quality of life [8–13].

To date, several interventions such as ICU diaries, early in-ICU psychological assessment, and ICU follow-up clinics appear ineffective to improve or prevent this psychological burden [13–17]. A possible explanation for this absence of effect might be the lack of understanding of patient’s needs, expectations and preferences in such circumstances. Although Granja et al. previously provided insights into several ICU-specific treatment determinants of fear resulting in psychological PICS, little is known about patient’s post-ICU treatment preferences to help them cope with stress, fear, and anxiety [18]. Despite that national and international guidelines on what kind of follow-up care should be offered are lacking, there is a trend of increased availability of ICU follow-up clinics observed [19–21]**.** The lack of understanding of patient’s post-ICU needs is pivotal to improve ICU follow-up care and to determine potentially helpful and effective interventions [22].

Hence, the primary aim of the present study was to assess the needs and wishes of patients to receive information to help them better grasp ICU treatment. In addition, we assessed the prevalence of psychological impairments and the health-related quality of life to underscore the importance of an effective treatment entity.

## Methods

### Setting and procedure

This was a multicentre, retrospective cross-sectional study in Dutch ICU survivors. The study was performed in four mixed medical-surgical ICUs of community hospitals providing secondary care with each ICU annually treating 500 to 1000 patients. This study was approved by the Medical Ethics Committees United (MEC-U), Nieuwegein, the Netherlands, and the need for written informed consent was waived.

### Participants

Patients ≥ 18 years old and mechanically ventilated ≥ 48 hours were eligible for inclusion. Patients were excluded if they were admitted to the ICU after elective surgery, were pregnant, did not speak the Dutch language or had a known history of dementia. Patients were screened for eligibility between October 2016 and January 2017 and the start of data gathering was set on 1 October 2016. Calculating back from this time point, patients were retrospectively classified into five different cohorts: 1 month (4–8 weeks), 6 months (26–30 weeks), 12 months (52–56 weeks), 2 years (104–108 weeks) and 2.5 years (130–134 weeks) after discharge from the ICU, respectively.

Before the start of data gathering survival status was checked in the hospital’s patient information system. In case of missing information regarding survival, the family physician was contacted before making the first phone call. Patients eligible for inclusion were contacted by telephone before sending the questionnaire. They were asked about their preference to receive the questionnaire; either a hardcopy by postal mail or a digital questionnaire by e-mail that was sent with an accompanying letter. Patients who expressed a wish not to take part during the phone call were excluded from further contact. In case of a non-response, patients were recontacted twice.

### Measures

A novel question set was designed to determine psychological PICS, HRQoL and the need for information regarding the ICU stay/treatment. This question set consisted of a combination of validated questionnaires (depression, PTSD, HRQoL) and a self-composed questionnaire regarding the needs and preferences of information (Additional file 1). In the latter part of the question set, patients were asked if they received information from their treating hospital, if they were satisfied with this information, if they had a desire to receive information, and if they had a desire to receive this information using a hardcopy information brochure or digitally. We also asked if patients had a wish to visit their ICU digitally so that they could re-experience their ICU stay, see their treatment environment and concurrently receive information about their treatment in this digital environment.

PTSD was assessed using the Impact of Event Scale Revised (IES-R) [23]. This questionnaire has previously been validated in survivors of critical illness [24]. The 22-items of the IES-R are rated on a 5-point Likert scale ranging from 0 through 4 (0 = not at all, 4 = extremely). A total cumulative score ≥ 33 is indicative for clinically relevant symptoms of PTSD [25].

Depression was measured using the Beck Depression Inventory (BDI). This questionnaire consists of 21 items representing symptoms of depression, which were scored on a 4-point Likert scale ranging from 0 through 3 [26, 27]. By combining the different items, the total BDI score can be calculated, ranging from 0 through 63, with scores > 13 suggesting clinically relevant symptoms of depression and scores < 28 suggesting severe depression [8, 28].

HRQoL was assessed with the EuroQol 5 dimensions questionnaire (EQ-5D-5L) and the Short-Form 12 (SF-12). The EQ-5D-5L is short, easy to use and shows good responsiveness in vulnerable patients [29]. The EQ-5D-5L measures the HRQoL on five dimensions (mobility, self-care, usual activities, pain/discomfort and anxiety/depression) which are evaluated within five severity levels (no problems, slight problems, moderate problems, severe problems, extreme problems) [30]. Subsequently, the weight of that health state is computed by a formula that firstly yields a partial weight score for each domain depending on the reported level and secondly adds the utility weight (also referred to as the ‘tariff’), which are based on the preference data of the general population of the Netherlands [31]. This score ranges from − 0.446 (worst quality of life) to 1.000 (best quality of life). Additionally, patients scored their current subjective health state on a visual analogue scale (EQ-VAS), ranging from 0 (worst health imaginable) to 100 (best health imaginable).

The SF-12 is an 8-scale profile of scores as well as physical and mental health summary measures: physical functioning (PF, two items), role limitations due to physical functioning (RP, two items), bodily pain (BP, one item), general health perception (GH, one item), vitality (VT, one item), social functioning (SF, one item), role limitations due to emotional problems (RE, two items), and mental health (MH, two items) [32]. Using the SF-12, the physical component scale (PCS-12) and the mental component scale (MCS-12) can be computed, with a mean of 50 and a standard deviation of 10 in the general population and giving a perception of a patient's’ mental and physical health state.

Socio-demographic characteristics were additionally asked within the questionnaire. Medical history and information about ICU treatment were assessed via digital patient records.

### Statistical analysis

All data was analysed using nonparametric tests to reduce the chance on a type I error. All continuous data is expressed as median (interquartile range/IQR). Categorical data was reported as absolute and relative frequencies, including, if appropriate, the 95% confidence interval.

Patients were stratified based on the presence of psychological PICS, defined as clinically relevant symptoms of PTSD and/or depression. PTSD was classified as an IES-R score above 33 and depression was classified as a total BDI score above 13 [25, 27]. HRQoL was determined using the EQ5D utility score.

To compare means of continuous variables between patients with and without psychological PICS, a Wilcoxon rank-sum test was used for continuous variables. To compare the differences in frequencies of categorical variables in patients with and without psychological PICS, a Fisher’s exact test was used.

The HRQoL of the entire population and of patients with and without psychological PICS was compared with HRQoL of the general Dutch population using a Standard Student’s *T* test [31].

For the association between the HRQoL and PTSD and depression, bivariate correlations were evaluated using a Spearman’s rho. A multivariate stepwise regression analysis with HRQoL (EQ-5D utility score) as a dependent variable and PTSD (total IES-R score) and depression (total BDI score) was performed to analyse the association between HRQoL and psychological PICS. Standardized regression coefficients with 95% CI were used to quantify the strength of the correlation within the regression model.

The results of the preferred intervention methods inventory are presented as absolute and relative frequencies on several items.

A *P* value < 0.05 was considered statistically significant. All analyses were performed using R for Statistics (R Foundation for Statistical Computing, Vienna, Austria, 2015).

## Results

**Fig. 1:**
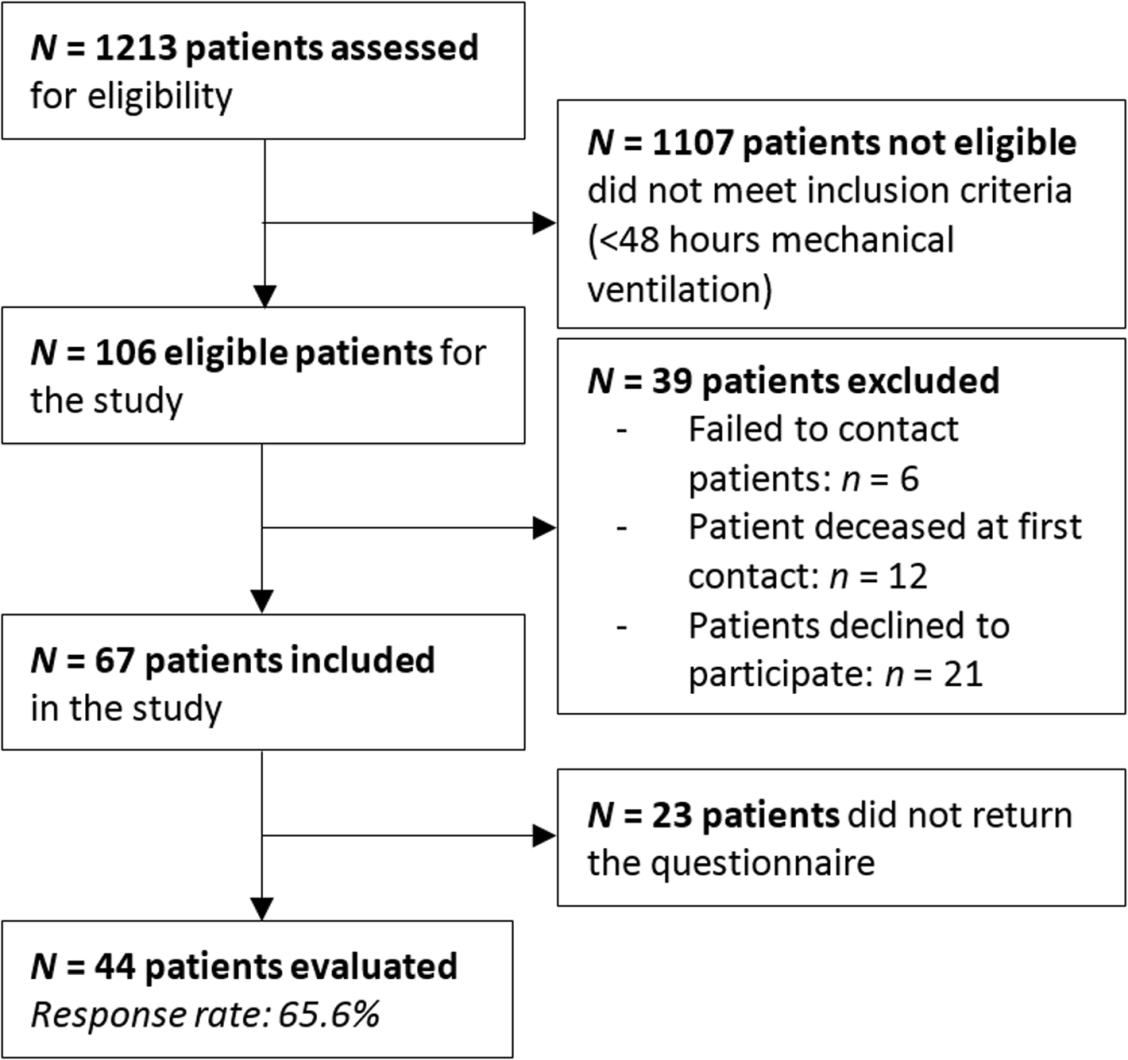
Flow diagram of the study

Of the 1213 patients who were screened for eligibility, 106 patients were found to be eligible. Of these eligible patients, 67 patients initially consented for participation and received the questionnaire. Finally, 44 patients completed and returned the questionnaire (response rate: 66%, see Fig. 1). Demographic and clinical characteristics are reported in Table 1. The median age was 61 years (IQR: 55–69, range: 22–76). Patients had a high severity of illness at ICU admission (median (IQR) APACHE II score, 21 (16–27); median (IQR) SAPS II score, 41 (32–55); median (IQR) admission SOFA score, 6 (5–9)). Median (IQR) ICU length of stay (ICU-LOS) was 4 (3–8) days and the median hospital length of stay was 15 (IQR: 10–22).

**Table 1:** Characteristics of patients at baseline

Characteristics		Total (*N* = 44)	Patients with psychological PICS (*N* = 26)	Patients without psychological PICS (*N* = 18)	*P* value†
Demographics					
Male, *n* (%)		35 (80%)	21 (81%)	14 (78%)	1.00^a^
Age, median (IQR)		61 (55–69)	59 (53–68)	64 (57–69)	0.20^b^
Treatment-related characteristics					
Hospital days, median (IQR)		15 (10–22)	14 (10–22)	15 (10–23)	0.90^b^
ICU days, median (IQR)		4 (3–8)	4 (3–8)	5 (3–8)	0.70^b^
Documented delirium, *n* (%)		9 (21%)	5 (19%)	4 (22%)	1.00^a^
Mechanical ventilation, h, median (IQR)		56 (30–132)	55 (28–123)	56 (33–153)	0.70^b^
Renal replacement therapy, *n* (%)		3 (7%)	1 (4%)	2 (11%)	0.60^a^
Scores					
APACHE II, median (IQR)		21 (16–27)	21 (16–26)	21 (16–21)	0.50^b^
SAPS II, median (IQR)		41 (32–55)	40 (32–54)	41 (32–54)	0.90^b^
Admission SOFA score, median (IQR)		6 (5–9)	7 (5–9)	6 (5–11)	1.00^b^
PTSD and depression					
IES-R score, median (IQR)		34 (24–46)	44 (36–56)	24 (22–25)	< 0.001^b^
BDI score, median (IQR)		10 (6–18)	16 (11–22)	3 (2–6)	< 0.001^b^
HRQoL					
SF-12	MCS-12, median (IQR)	48 (39–55)	40 (36–45)	56 (54–59)	< 0.001^b^
	PCS-12, median (IQR)	40 (35–46)	38 (34–45)	42 (37–47)	0.30^b^
EQ-5D utility score, median (IQR)		0.75 (0.53–0.88)	0.68 (0.45–0.82)	0.88 (0.76–0.98)	< 0.01^b^
EQ-VAS, median (IQR)		65 (54–80)	58 (45–68)	70 (66–84)	< 0.01^b^

*PICS* post-intensive care syndrome, *PTSD* posttraumatic stress disorder, *IES-R* Impact of Event Scale Revised, *BDI* Beck Depression Inventory, *HRQoL* health-related quality of life, *SF-12* Short-Form 12, *MCS-12* Mental Component Scale from the SF-12, *PCS-12* Physical Component Scale from the SF-12, *EQ-5D* EuroQol 5 Dimensions, *VAS* visual analogue scale

^†^*P* values are for the comparison between patients with and without psychological PICS

^a^*P* value from Fisher’s exact test

^c^*P* value from Wilcoxon rank-sum test

### Posttraumatic stress disorder and depression

**Fig. 2:**
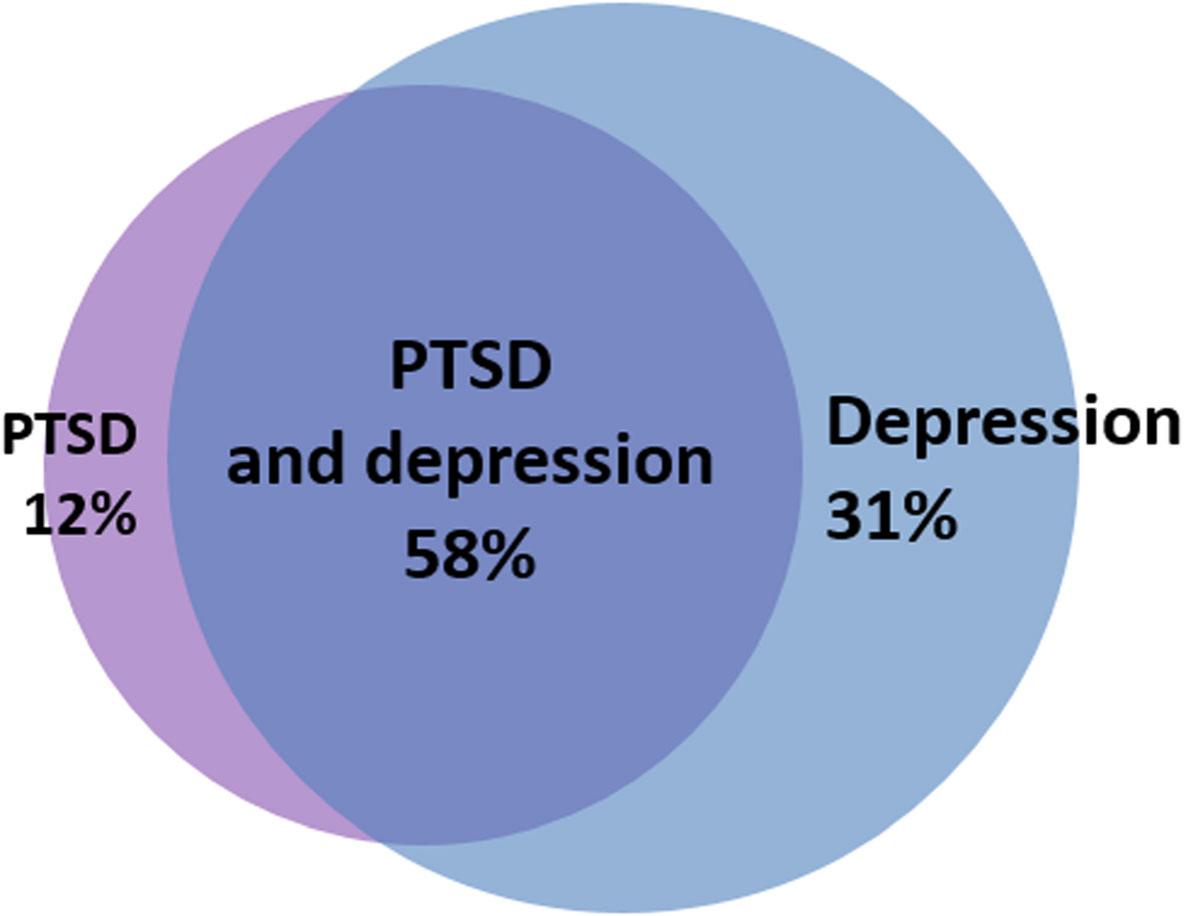
Venn-diagram of the occurrence of PTSD and depression within patients with PICS

Twenty-six (59%, 95% CI 44% to 74%) patients suffered from psychological PICS. These patients had both significantly more symptoms of PTSD (median (IQR) IES-R score, 44 (36–56) vs. 24 (22–25), patients with psychological PICS vs. patients without psychological PICS, *P* < 0.001) and significantly more symptoms of depression as patients without psychological PICS (median (IQR) BDI score; 16 (11–22) vs. 3 (2–6), *P* < 0.001). Three (12%) patients with psychological PICS suffered solely from PTSD, 8 (31%) solely from depression and 15 (58%) suffered from both PTSD and depression, as shown in Fig. 2.

The prevalence of psychological PICS was similar in patients over time. Additionally, psychological PICS was present in 6 out of 10 (60%, 95% CI 30% to 90%) patients after 1 month, 7 out of 10 (70%, 95% CI 42% to 98%) after 6 months, 4 out of 8 (50%, 95% CI 15% to 85%) after 12 months, 6 out of 9 (67%, 95% CI 36% to 98%) after 24 months and 3 out of 7 (43%, 95% CI 6% to 80%) patients after 30 months.

### Preferred intervention methods

Twenty-one out of 43 (49%) patients received an information brochure, of whom 14 (67%) expressed a wish to receive additional information regarding their ICU treatment. Of the 22 patients who did not receive any information from their treating hospital, 17 (77%) expressed a wish to receive information regarding their ICU treatment. Of the 31 patients who had a wish to receive information, 22 (71%) had a desire to receive this information from an ICU nurse and/or an intensivist.

Looking at the complete cohort, only 21 (49%) patients were satisfied with the information received. The most mentioned reason for not desiring information was the time past between ICU treatment and the questionnaire. Although only 13 (30%) patients were already familiar with Virtual Reality, 26 (60%) thought VR would be valuable for delivering this information after receiving a written explanation about the techniques and possibilities of VR.

Nine out of 25 patients with psychological PICS received information using an information brochure. From these patients, 6 (67%) expressed a wish to receive additional information. Of the 16 patients who did not receive any information, 13 (81%) expressed a wish to receive information regarding ICU treatment. Only 8 (33%) of all patients with psychological PICS were satisfied with the information received. Thirteen (54%) patients with psychological PICS thought VR could be valuable to deliver information; additionally, 4 (17%) patients thought it could be valuable but thought it would be too frightening to actually undergo VR exposure. Only 7 (29%) patients thought VR would not be valuable to ameliorate psychological recovery.

### Quality of life

**Fig. 3:**
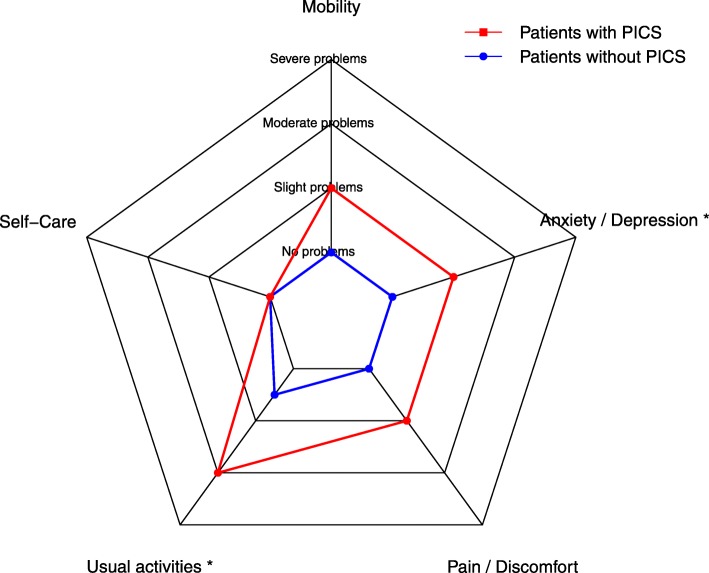
Radar chart of outcomes of the EQ-5D by presence of psychological PICS. *Legend*: Spider plot of the outcomes of the domains of the EQ-5D by presence of PTSD and/or depression (psychological PICS), presented as means. *P* values were calculated using a Wilcoxon Rank Sum Test. * *P* < 0.01

Quality of life was classified using the EQ-5D questionnaire. Problems of any severity on the domain of usual activities were reported by 32 patients (73%) in the overall population, problems on the domain of pain/discomfort by 26 patients (59%), problems on the domain of mobility by 23 patients (52%), problems on the domain of anxiety/depression by 22 patients (50%) and problems on the domain of self-care by 16 patients (36%) (see Table 2). Patients with psychological PICS scored significantly worse on the domains of usual activities (*P* = 0.01) and anxiety/depression (*P* = 0.01) (Table 2, Fig. 3).

**Table 2:** EQ-5D descriptive system results by presence of psychological PICS

Category	Severity	Total (*N* = 44)	Patients with psychological PICS (*N* = 26)	Patients without psychological PICS (*N* = 18)	*P* value*
Mobility	No problems, *n* (%)	21 (48)	11 (42)	11 (56)	0.70
	Slight problems, *n* (%)	8 (18)	6 (23)	2 (11)	
	Moderate problems, *n* (%)	8 (18)	5 (19)	3 (17)	
	Severe problems, *n* (%)	6 (14)	4 (15)	2 (11)	
	Unable, *n* (%)	1 (2)	0 (0)	1 (6)	
Self-care	No problems, *n* (%)	28 (64)	14 (54)	14 (78)	0.30
	Slight problems, *n* (%)	8 (18)	5 (19)	3 (17)	
	Moderate problems, *n* (%)	3 (7)	3 (12)	0 (0)	
	Severe problems, *n* (%)	3 (7)	3 (12)	0 (0)	
	Unable, *n* (%)	2 (5)	1 (4)	1 (6)	
Usual activities	No problems, *n* (%)	12 (27)	3 (12)	9 (50)	0.01
	Slight problems, *n* (%)	16 (26)	9 (35)	7 (39)	
	Moderate problems, *n* (%)	11 (25)	10 (38)	1 (6)	
	Severe problems, *n* (%)	2 (5)	2 (8)	0 (0)	
	Unable, *n* (%)	3 (7)	2 (8)	1 (6)	
Pain/discomfort	None, *n* (%)	18 (41)	7 (27)	11 (61)	0.07
	Slight, *n* (%)	0 (0)	0 (0)	0 (0)	
	Moderate, *n* (%)	12 (27)	7 (27)	5 (28)	
	Severe, *n* (%)	11 (25)	9 (35)	2 (11)	
	Extreme, *n* (%)	3 (7)	3 (12)	0 (0)	
Anxiety/depression	None, *n* (%)	22 (50)	8 (31)	14 (78)	0.01
	Slight, *n* (%)	15 (34)	12 (46)	3 (17)	
	Moderate, *n* (%)	3 (7)	2 (8)	1 (6)	
	Severe, *n* (%)	4 (9)	4 (15)	0 (0)	
	Extreme, *n* (%)	0 (0)	0 (0)	0 (0)	

*PICS* post-intensive care syndrome, *EQ-5D* EuroQol 5 dimensions

^*^P value from Fisher’s exact test for the comparison between patients with and without psychological PICS

Patients in our cohort as a whole reported a worse HRQoL compared to the general Dutch population (mean difference = − 0.17, 95% CI − 0.25 to − 0.10, *P* < 0.001). Moreover, patients with psychological PICS had a significantly worse HRQoL compared to the general Dutch population (mean difference = − 0.27, 95% CI − 0.38 to − 0.16, *P* < 0.01), in contrast to patients without psychological PICS (mean difference = − 0.04, 95% CI − 0.12 to 0.04, *P* = 0.30). Subsequently, HRQoL was lower for patients with psychological PICS compared to patients without psychological PICS (median (IQR): 0.68 (0.45–0.82) vs. 0.88 (0.76–0.98), *P* < 0.01). The subjective health state as scored using the EQ-VAS score was significantly lower for patients with psychological compared to patients without psychological PICS (median (IQR): 58 (45–68) vs. 70 (66–84), *P* < 0.01).

Furthermore, the mental component scale measured within the SF-12 (MCS-12) was significantly lower in patients with psychological PICS (median (IQR): 40 (36–45) vs. 56 (54–59), *P* < 0.001), while the physical component scale (PCS-12) did not differ between patients with and without psychological PICS (median (IQR): 38 (34–45) vs. 42 (37–47), *P* = 0.30).

### Association between HRQoL and psychological PICS

A significant association was found between the severity of PTSD (total IES-R score) and severity of depression (total BDI score; Spearman’s ρ = 0.712, *P* < 0.001). There was a significant association between patients’ HRQoL and both the severity of PTSD (total IES-R score; Spearman’s ρ = − 0.531, *P* < 0.001) and the severity of depression (total BDI score; Spearman’s ρ = − 0.643, *P* < 0.001).

## Discussion

In the current study, we investigated whether the needs, expectations and wishes of patients were met using the commonly used information brochure or whether patients preferred alternative delivering methods to better grasp ICU treatment. In addition, we assessed the prevalence of psychological impairments, the HRQoL and its association in a cohort of ICU survivors.

Our data underscores that psychological post-ICU sequelae, such as PTSD and depression, are a major clinical concern that may persist for several years after ICU discharge and is associated with a considerable decrease in HRQoL. Before routine follow-up of ICU-patients with such chronic conditions can be successful, it is important to focus on the unmet healthcare needs of these patients. The current results clearly demonstrate that patients suffering from psychological PICS have a self-reported unmet healthcare need of information about their treatment. Subsequently, they are more interested in receiving digital information about their ICU stay and treatment using a video film or VR compared to the currently accepted hardcopy information brochure. This is the first study that demonstrated the patient’s wish for information regarding ICU treatment and that a video or VR film might be a valuable adjunct.

Apart from these findings, the combination of questionnaires in the current study, which enables patients to give voice to their experiences from 1 month to 2.5 years after ICU discharge, is a novel feature of self-reported unmet healthcare needs. This adds to our understanding on how patients make sense of what has happened to them and what they need to confront their fear after ICU-related trauma. Moreover, the current cohort demonstrated that the prevalence of psychological PICS persists over time, up to 2.5 years. This is in line with recent findings by Bienvenu et al. demonstrating that symptoms of anxiety, depression and/or PTSD are common in the first 5 years after critical illness and has a similar incidence over time. Our findings go beyond describing the incidence of PICS in our cohort but clearly give a patient’s opinion on the preference of needs and wishes on post-ICU interventions. To date, knowledge about the needs of patients suffering from PICS are scarce [33], and an effective treatment and thereby a uniform aftercare protocol is missing for patients suffering from PICS. Our findings can therefore be used to develop new treatment strategies, which can be implemented in an aftercare protocol in order to ameliorate the HRQoL of these patients.

A recent survey of ICUs in the Netherlands demonstrated that the majority of ICU’s evaluate health status and restrictions in functioning after ICU treatment [19]. Hence, 61% of the hospitals has or currently is developing ICU follow-up care. There is a high probability that this number is even higher because the survey was performed in 2014. This percentage is in accordance to the situation in the UK and the USA. To date, no study has identified generalizable mechanisms by which post-ICU programs could systematically treat psychological sequelae. It is therefore not surprising that several interventions like ICU diaries [14, 17], ICU follow-up clinics [13, 34] or a primary care-focused team-based intervention [16] did not have a significant effect to improve or prevent the psychological burden nor improving health-related quality of life. A recent study by Heydon et al. demonstrated that patients with psychological PICS related impairments identify these complaints as the most important area where they want support in [35]. Additionally, psychological PICS is referred to as the most important component of patient-reported unacceptable outcome by a recent study by Kerckhoffs et al. [7]. We confirm these findings by demonstrating that PTSD and depression are both associated with a considerable decrease in quality of life and that patients with psychological PICS have a worse quality of life compared to the general Dutch population in contrast with patients without psychological PICS. This is in line with a previous study by Wang et al., who demonstrated that the comorbidity of psychiatric symptoms is associated with a worse quality of life [33]. To improve the success of post-ICU clinics, treatment of psychological sequelae is critical to improve HRQoL. Additionally, our data suggest that mental health is a more important contributing factor to decreased health-related quality of life compared to the physical component. The mental health-related quality of life (MCS12) was decreased in patients with psychological PICS, whereas the physical health-related quality of life (PSC12) was comparable for patients with and without psychological PICS.

Psychological impairments in a post-ICU population may be understood as a consequence of amnesia during their early period of critical illness state. This leads to loss of factual recall and instead creates delusional memories that are the first to return [18, 36, 37]. Both mechanisms lead to a false recollection of the ICU stay resulting in anxiety and PTSD related symptoms [18, 37]. Changing how patients visually and auditively experienced the ICU and truly understand their ICU treatment may therefore be an important step towards recovery. Although an information brochure is able to reduce stress symptoms of relatives, loss of factual recall may explain why patients are not interested in such a brochure. An information brochure is not able to visually and auditively address amnesia. This might explain why the majority of patients were motivated to undergo a video/VR experience to help them address their questions. Compared to a flatscreen video, VR is a new interactive and immersive technology that makes it possible to reinforce the human connection in an immersed technological environment [38]. Subsequently, several recent studies demonstrated beneficial effects of VR in the treatment of several mental health disorders, including PTSD and anxiety [39–45]. It can therefore be hypothesized that a VR based intervention might also be of additional value to stimulate patients after ICU treatment suffering from psychological PICS [46–48]. Due to the current technological improvements and lowering in costs, an ICU-specific VR video is worthy of further investigation [49].

The current study has several limitations that should be acknowledged. First, patients received treatment several months (in some cases even years) earlier and we therefore would expect some recall bias. This is however also partly the problem as fragmented (delusional) memories make it extremely difficult for patients to create understanding of their ICU treatment [50]. In its turn, a natural selection of patients that were willing to participate could have been created due to the retrospective nature. It could be hypothesized that patients for whom ICU-related thoughts could be too intrusive, declines to participate and results in selection bias. Despite this hypothesis, 63% (67 patients) of the eligible patients initially consented to participate, and 41% (43 patients) of eligible patients did return the questionnaire. This is twice as much when compared to similar studies in emergency department patients [51]. Second, the questions were not mandatory to answer, which could have increased bias. However, only 1 (4%) patients did not answer the questions regarding the preferred intervention methods and all participants answered all other questionnaires. Last, the interpretation of the outcomes of statistical analyses in this manuscript is hampered by the small samples size. As such, clinical translation of the effect sizes that we exhibit in this study is more meaningful than solely focusing on the reported *P* values. We believe that in the current study, the statistically significant results are in line with clinically relevant effect estimates. Moreover, due to the small sample size, we choose to analyse patients as one cohort.

## Conclusions

In a cohort of critical illness survivors, patient suffering from psychological PICS are in need of information, have no desire using an information brochure but are willing to receive information via digital content such as a video film/VR. Conceptualizing patient experiences and treatment understanding might therefore be a well-appreciated new strategy to help patients cope with there (delusional) memories, problems and questions. These results lay the groundwork for developing such interventions to be tested in post-ICU programs and to determine whether mental health can be improved.

## Supplementary information

**Additional file 1.** Preferred intervention method inventory.

## Data Availability

The datasets analysed during this study are available from the corresponding author for a reasonable request.

## Abbreviations

95% CI: 95% confidence interval
BDI: Beck Depression Inventory
EQ-5D-5L: EuroQol 5 Dimensions 5 Levels
EQ-VAS: EuroQol Visual Analogue Scale
HRQoL: Health-related quality of life
ICU: Intensive care unit
IES-R: Impact of Event Scale Revised
IQR: Interquartile range
MCS-12: Mental Component Scale, measured using the SF-12
PCS-12: Physical Component Scale, measured using the SF-12
PICS: Post-intensive care syndrome
PTSD: Posttraumatic stress disorder
SD: Standard deviation
SF-12: Short-Form 12
VR: Virtual reality
