# Supplementary material for: Patients suffering from psychological impairments following critical illness are in need of information
Source: J Intensive Care. 2020 Jan 9;8:6. doi: 10.1186/s40560-019-0422-0 (PMC6953141; doi:10.1186/s40560-019-0422-0)
Supplement: Supplementary file 1 — Additional file 1. Preferred intervention method inventory. [file 40560_2019_422_MOESM1_ESM.docx]

# Preferred intervention inventory

The following 9 questions are about applications we could possibly implement in the future to ameliorate the psychological recovery from the Intensive Care.

1. Have you received an information brochure about your stay and treatment in the Intensive Care Unit?

□ Yes

□ No

If you answered ‘Yes’, was an information brochure your preferred method to receive this information?

□ Yes

□ No

If you answered ‘No’, did you have a desire to receive information using an information brochure?

□ Yes

□ No

1. Are you satisfied with the explanation and amount of information you received after your ICU treatment?

□ Yes

□ Yes, but I’m still wondering about certain aspects

□ Yes, I understand how my disease was treated, but not how everything on the ICU works

□ Yes, but I would have like to look digitally further into certain aspects

□ No

1. Is it, in your opinion, valuable to provide additional information to patients about their stay in the ICU and what is happening during ICU treatment (i.e. explanation about sounds and noises, machines in the ICU room, monitors in the ICU room)

□ Yes

□ No

□ I don’t know

1. Do you have a desire to receive an one-off explanation about everything that’s happening on the ICU from a ICU nurse/intensivist?

□ Yes, by an ICU nurse

□ Yes, by an intensivist

□ No, I would like to receive explanation multiple times

□ No, I prefer an information video so that I can look up information and watch it multiple times

□ No, other reason:___________________________________

1. Are you familiar with Virtual Reality?

□ Yes

□ No

*Virtual Reality is a technique that immerses persons into a virtual environment. It is mostly used via VR-glasses. By delivering a slightly different image to both eyes, users get the feeling they are in the shown environment, rather than the environment they are actually in.*

*Virtual Reality can be used to expose a person to a certain environment, with the additional option to offer information. Virtual Reality could therefore be used to revisit the Intensive Care virtually and offer information about ICU treatment.*

1. Would you desire to use Virtual Reality to get a better understanding about the ICU treatment?

□ Yes, I would like to see how the ICU room looks like through VR

□ Yes, I’m still wondering how the rest of the department looks like

□ Yes, I think that if I would have had the chance to get familiar with the ICU environment and would have received proper explanation, that the treatment would have been less heavy

□ Yes, other reason

□ No, I would be too afraid to do so

□ No, I would not

1. If you have to make a choice between the different manner of delivering additional information, which one would be, in your opinion, the most valuable? You can choose multiple answers.

□ An information brochure

□ An information video

□ A VR-experience
